# Structure, Merits, Gel Formation, Gel Preparation and Functions of Konjac Glucomannan and Its Application in Aquatic Food Preservation

**DOI:** 10.3390/foods12061215

**Published:** 2023-03-13

**Authors:** Yilan Sun, Xiaowei Xu, Zhenzhen Wu, Hanlin Zhou, Xiaoyu Xie, Qinhua Zhang, Renyi Liu, Jie Pang

**Affiliations:** 1 Center for Agroforestry Mega Data Science, Haixia Institute of Science and Technology, Fujian Agriculture and Forestry University, Fuzhou 350002, China; 2 College of Life Sciences, Fujian Agriculture and Forestry University, Fuzhou 350002, China; 3 College of Food Sciences, Fujian Agriculture and Forestry University, Fuzhou 350002, China

**Keywords:** konjac glucomannan, gel formation, food preservation, aquatic food

## Abstract

Konjac glucomannan (KGM) is a natural polysaccharide extracted from konjac tubers that has a topological structure composed of glucose and mannose. KGM can be used as a gel carrier to load active molecules in food preservation. The three-dimensional gel network structure based on KGM provides good protection for the loaded active molecules and allows for sustained release, thus enhancing the antioxidant and antimicrobial activities of these molecules. KGM loaded with various active molecules has been used in aquatic foods preservation, with great potential for different food preservation applications. This review summarizes recent advances in KGM, including: (i) structural characterization, (ii) the formation mechanism, (iii) preparation methods, (iv) functional properties and (v) the preservation of aquatic food.

## 1. Introduction

Konjac glucomannan (KGM) is a hydrophilic biomolecule derived from konjac tubers and consists of glucose and mannose linked by β-1,4-glucosidic bonds [1,2]. KGM can be used as a stabilizer or a gelling and thickening agent in the food industry due to its excellent properties in gel-forming, film-forming and water-holding capacity [3]. KGM gel types are made in different forms, including liquid gels, aerogels and solid gels [4,5,6], with the liquid gels being the most widely used. Inhibitors, adsorbents and adhesives are developed based on the characteristics of KGM liquid gels, such as bioactivities, biocompatibility and swelling properties [7]. Under plus force, the molecular groups of KGM can form topological structures by bonding with reactive molecules containing hydroxyl, thereby enhancing the stability of the reactive molecules [8]. The resulting structurally stable gel system serves the purpose of food preservation by providing protection to the unstable antioxidant active molecules.

Aquatic food includes finfish, crustaceans, cephalopods, other mollusks, aquatic plants, algae and other aquatic animals [9]. In comparison to other muscle foods, aquatic foods are prone to quality deterioration caused by various factors, such as physiological and biochemical decay, lipid oxidation, damage from endogenous enzymes and infections by spoilage and pathogenic microorganisms [9,10]. Their quality also tends to deteriorate quickly after capture and further during storage when compared to fruits and vegetables. To address these challenges, various preservation methods have been developed [9]. These methods can be broadly categorized based on their impact on human health. Physical methods, such as irradiation, ultraviolet light, pulsed light, cold plasma, high-pressure processing, ultrasound, temperature management and modified atmosphere packaging techniques, are less hazardous but can potentially cause structural damage to the food [11,12,13]. Chemical preservation techniques, such as chlorine, bromine, iodine, trisodium phosphate and organic acids, offer a long shelf-life but can pose health risks due to a lack of regulation and potential overuse [14,15]. Consequently, alternative preservation methods that are safer, more effective, cost-effective and environmentally friendly are needed. One such solution is the preservation of aquatic products using antioxidant active molecules [16]. However, ensuring the stability of these substances is critical, as they are susceptible to oxidation and inherently unstable.

Various biopolymers, including polysaccharides such as starch, gum, alginates, chitosan and carrageenan, have been used in biological treatments for food preservation, and KGM is an excellent source of polysaccharides [15,17,18,19]. As a highly stable natural polymer compound, KGM has a strong water-holding capacity due to the presence of a large number of hydrophilic groups in its molecular structure. After dissolving in water, the dense network structure of KGM becomes an effective barrier that facilitates the encapsulation, protection and sustained release of active molecules, such as polyphenols, nanoparticles and marine polysaccharides [20]. Moreover, these active substances can form a robust structure with KGM through chemical bonding to prevent microbial invasion, and they can be used for food preservation [21]. Despite these advantages, there is currently no systematic review available that comprehensively covers the application of KGM gels in aquatic foods.

In this review, we present the structural characterization and merits of KGM, followed by a discussion of the formation mechanism and preparation methods of KGM gel. We also illustrate the functional properties of KGM gel when loaded with active substances and recent developments related to its use in the preservation of aquatic foods. This article aims to shed light on the potential of KGM gels in promoting the development of high-quality aquatic food products.

## 2. Konjac Glucomannan and Its Structural Features 

### 2.1. Sources

Konjac is a root vegetable belonging to the Araceae family and is widely cultivated in the mountains of China, Japan and Southeast Asia [22]. KGM is a natural soluble polysaccharide obtained from the konjac tuber, with a unique hydrogen bond network, spiral structure and topological structure [22].

### 2.2. Structure

#### 2.2.1. Hydrogen Bond Network

The hydrogen bond network structure of KGM refers to the chain, ring, layered or three-dimensional network structure formed by the hydrogen bond interactions between KGM molecules. The hydrogen bond network structure gives KGM molecules strong cohesive forces, making them difficult to separate, even at high temperatures. The hydroxyl groups form hydrogen bonds with functional groups in KGM, resulting in a more compact network structure than that of pure KGM gels [23]. Pang et al. investigated the effect of different conditions (temperature, pH value, urea) on the hydrogen bonding network of KGM and the effect of inorganic molecules on the hydrogen bonding network and properties of KGM [24,25,26,27,28]. The results showed that the conformation of KGM after dissociating acetyl and at pH = 11, with more hydrogen bonds and lower energy, was more stable. The urea solution decreased the stability of KGM. All details are presented in Table 1 [24,25,26,27,28]. In conclusion, alterations in the hydrogen bonding network greatly impact the stability of the KGM conformation, ultimately leading to changes in the gelation properties of KGM.

#### 2.2.2. Helical Structure

The helical structure of polysaccharides is essential for their properties and biological activities, as they can potentially be used as carriers for various functional polynucleotides [29]. Previous studies on KGM’s helical structure (as shown in Table 2) revealed that the overall conformation of KGM molecules is a regular α-helical structure [30]. There is a uniform and stable hydration layer between the left-handed single-helical molecular chain and water molecules, while the right-handed single-helical molecular hydration shell has interrupted distribution at many sites [25]. These findings suggest that the entire glucan macromolecular chain structure is composed of stable local left-handed and right-handed helices [31].

The helical structure of KGM molecules may be influenced by the degree of deacetylation, the degree of polymerization and the temperature. The partial removal of acetyl groups in KGM may change the morphology of the KGM molecular chain by affecting the distribution of hydrogen bonds within the molecule [32]. As the degree of aggregation or temperature increases, KGM molecular chains show an irregular helical state and lower stability [33]. Additionally, temperature has a destructive effect on the helical structure, but it is reversible [34]. At 341 Kelvin, the helical structure disappears and turns into a random nematic structure, which can partially be restored with the decreased temperature [30]. Thus, the helical structure can be adjusted by the above factors.

**Table 2:** The research progress of the KGM helical structure [26,30,35].

Object	Research Contents	Result	Method	Reference
KGM chain of vacuum	Influence of the degree of polymerization, acetyl group and nonbonding force on the chain morphology	Acetyl is important in maintaining the helical structure of the KGM molecule; the degree of polymerization of KGM significantly affects the shape and stability of its helical structure	Molecular dynamics	[30]
Unbranched KGM	KGM helix formation site, helix parameters and hydrogen bond sites	The KGM molecular chain may form a helix on the segments containing acetyl groups	Computer simulation	[35]
KGM mono-helix	Local maximum water density near the KGM single-helix	The left-handed single helical conformation is the dominant conformation of KGM in an aqueous environment	Molecular dynamics	[26]

#### 2.2.3. Topological Structure

The KGM topology describes the molecular composition of KGM in mathematical terms, reflecting the way in which KGM molecules are connected. With the topological equation of KGM’s molecular conformation parameters, the spatial structure changes of molecular chains can be explained, allowing for the regulation of the KGM molecular structure [36]. The typical topological “8” type cross-linked ring can enhance the mechanical properties of macromolecules and create a stable network structure, providing thermal stability, even under heating conditions [37,38]. KGM molecules are primarily linked by hydrogen bonds within and between the molecules. The main chains are connected to form a circular topology, while the side chains are linked to each other through their OH groups, making the circular topology structure more robust [39]. As a result, the topological structure formed by KGM can serve as a stable carrier for loading natural active ingredients.

The current research progress on KGM topology is shown in Table 3 [40,41]. The topological structure that KGM forms can improve its stability, thus providing protection to the loaded natural active substances with a high release rate [8]. 

### 2.3. Merits of KGM for Aquatic Food Preservation

KGM possesses a range of favorable properties, such as film-forming, gelation, water retention and thickening properties, which make it suitable for the preservation of aquatic foods. Compared to traditional packaging, KGM films offer several advantages, including safety, environmental protection and biodegradability.

#### 2.3.1. Film Forming

KGM has a high degree of interchain bonding that makes the polymer insoluble in water [42]. However, KGM requires a large amount of water to swell adequately, limiting the concentration of KGM gel and affecting its film-forming ability and strength [43]. After dehydration, KGM can be made into a high-transparency and high-density film that remains stable for several hours in cold or hot water, and even in boiled acid. Additionally, KGM films are edible, naturally degradable and can be used as coated films for the preservation of aquatic food or for overwrapping [44].

#### 2.3.2. Gelation

In alkaline solutions, KGM chains lose acetyl groups and form a gel network through hydrogen bonding. Gelation is improved with lower acetylation degrees and higher hydroxyl functional groups [2,45]. The alkaline method is the most widely used for KGM gel preparation [46]. Increasing the degree of deacetylation achieves a faster gelation rate and a larger elastic modulus. Under heating or alkaline conditions, self-polymerization caused by deacetylation transforms KGM from a sol to a robust, elastic and thermally irreversible gel [47,48]. 

The three-dimensional gel network structure produced by KGM gelation is an excellent carrier for small active molecules and can be used to load antioxidant molecules for aquatic food preservation [49]. To further improve the stability and functionality of the gel carrier, researchers typically incorporate other biomacromolecules, nanoparticles and antioxidant and antibacterial substances to enhance gel performance [1].

#### 2.3.3. Water Retention

Water retention in KGM measures the ability to maintain water during freezing, making it an indicator for freeze–thaw stability. Two parameters constitute water retention: the water separation rate and water holding rate. The former quantifies the ability of KGM gels to resist shrinkage during freezing, while the latter reflects their capacity to retain water after high-speed centrifugation [50,51].

KGM particles are amorphous in structure and aggregate with water molecules through hydrogen bonding, molecular dipole, instantaneous dipole and induced dipole forces to form fixed macromolecules [48]. These particles are readily dissolved in water and exhibit a significant expansion in volume upon water absorption [52]. During the dissolution process, the diffusion migration rate of water molecules is higher than that of KGM, resulting in the swelling of KGM and an increase in water absorption [53]. When combined with water molecules through freezing, KGM forms more stable macromolecules and exhibits viscosity, making it a suitable gel carrier for aquatic food preservation [54,55].

#### 2.3.4. Thickening

Thickening is a process in which solid particles suspended in a liquid become a thick, concentrated slurry and are separated from the liquid. During storage, the slurry concentration will increase due to diluents or chemical reactions [48]. Because of its good swelling properties and high water absorption, KGM has a higher viscosity than other thickeners (e.g., carrageenan and gum Arabic) at the same concentration [56]. 

However, because KGM has a low solubility, a low stability of hydrosol and a lazy flow property, the utilization of KGM as an auxiliary thickener in aquatic food preservation has many restrictions. The grafting polymerization of water-soluble commoners (e.g., acrylamide) onto KGM is a good way to improve its thickening [57]. KGM molecular chains can intertwine with macromolecular chains through hydrogen bonding and van der Waals forces and form a three-dimensional mesh structure that generates a synergistic effect, especially when combined with carrageenan, xanthan gum or guar gum [32,58]. The stabilizing and thickening abilities of KGM are essential for its wide application with other natural biomacromolecule-based gels in aquatic foods preservation, packaging, drilling, coating, pharmaceuticals and cosmetics [59,60]. 

## 3. Formation Mechanisms of KGM Gels

### 3.1. Self-Assembly/Conjugate Structure

Self-assembly is a process by which smaller KGM molecules spontaneously come together to form larger, ordered aggregates. Hydrophobically modified KGM can act as amphiphilic molecules and self-assemble into spherical micelles in water. Amphiphilic block copolymers, which consist of hydrophilic and hydrophobic chain segments, are capable of non-covalent bonding in an aqueous solution through intra- and inter-molecular interactions [61]. As a result, micelles or micelle-like aggregates can be formed, with the hydrophobic chain segments forming the core and the hydrophilic chain segments forming the shell [62,63]. Self-assembly relies on the molecules’ ability to organize themselves into a well-defined pattern and aims to achieve a stable structure.

### 3.2. Chain Coupling Perforation

The macromolecular chains are interspersed to form a “chain-coupled perforated” structure, which contributes to its thermal stability [64]. This “chain-coupled perforation” forms a stable skeleton, and the relationship between the coupled perforation and the topological entanglement skeleton can be explored using multi-scale simulations, mathematical modeling and rheology and spectroscopy techniques. Relevant research provides insight into the mechanism of KGM stability regulation and the dynamic relationships between the conformation and properties of polysaccharide chains [65]. The macromolecular chains interweave to form a “wrapping” topological structure, ultimately forming a stable “chain-coupling perforation” structure, revealing the mechanism of KGM stability regulation and enhancing the dynamic relationship between the conformation and properties of the polysaccharide chains, thereby improving thermal stability [65].

### 3.3. Combination Mechanism of “Polyphenol-Embedded Topological Protection” 

KGM is a linear molecule with multiple groups on its surface. Analytical methods of knots are used for the analysis of the KGM topological structure because the complex KGM structure can be transformed into a straightforward planar topological structure [66,67]. Tea polyphenol (TP), a major constituent of tea, exhibits good biological activity and pharmacological effects [68]. Epigallocatechin gallate (EGCG) is the main component of TP, and it can combine with KGM molecules through hydrogen bonding. KGM chain-coupled perforation can identify the mosaic topological protection mechanism of TP. Using electrospinning technology, TP and KGM can be combined to form a mosaic topological gel, which efficiently protects the active molecules and improves their stability [69,70]. The combination of KGM and TP forms an embedded topological protection structure that enhances the stability of polyphenols and provides a theoretical basis for the preservation of aquatic foods. 

## 4. Preparation of a KGM Gel Carrier Loaded with Active Molecules

### 4.1. Casting Method

**Figure 1:**
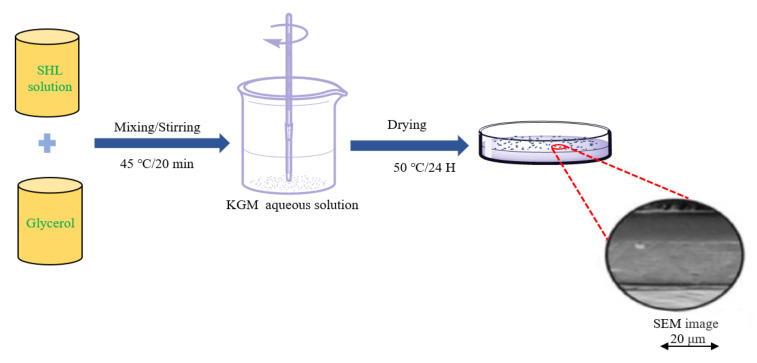
Scheme of the preparation of KGM/shellac films via casting (Adopted from Ni et al.) [71]. A shellac (SHL) solution and glycerol were added to the prepared KGM dispersion and stirred at 350 rpm for 20 min at 45 °C. Then, the mixed solution was slowly poured into a glass dish and placed in a vacuum drying oven at 50 °C for one day.

The casting method is a widely used technique for preparing KGM-based films for food preservation due to its simplicity, low cost and convenience [71,72,73,74,75,76,77,78,79,80,81]. To prepare KGM-based films by the solvent casting method, a film-forming solution is poured onto a substrate, followed by the natural volatilization of the solvent and the solidification of the film. A common scheme for this method is presented in Figure 1 [71]. Film formation occurs primarily through intermolecular and intramolecular hydrogen bonding interactions. While this method is simple, it can be challenging to control the uniformity of the film-forming solution, separate the films from the matrix and prevent bubble formation during the film formation process [82].

### 4.2. Microfluidic Spinning Technology (MST)

**Figure 2:**
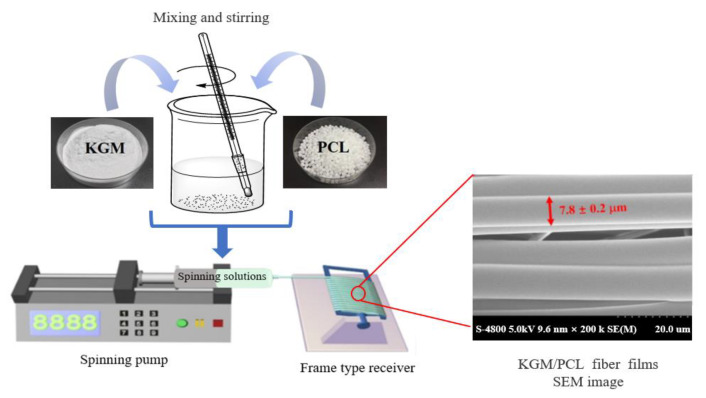
The formation process of producing KGM/poly(caprolactone) fiber films via microfluidic spinning (Adopted from Lin et al., Ni et al. and Lin et al.) [84,85,86]. Syringe pump speed of 0.5 mL/h, frame receiver speed = 400 arc/min and frequency of the horizontal stepping process = 25 Hz. KGM/ poly(caprolactone)(PLC) and PLC/Ag nanoparticle fibrous films were prepared under the same operating conditions.

Compared to the casting method, the electrospinning method (MST) is a promising technique for preparing polysaccharide-based films due to its flexibility and environmentally friendly features [83]. An example of the formation process is presented in Figure 2. The electrospinning method allows for the production of micro-fibrous films without the need for high-voltage electric fields or auxiliary measures [84,85]. In particular, it has shown great potential for fabricating KGM-based films through fluid interactions. However, the poor tensile strength of KGM requires the use of spinning aids during film preparation, despite the possibility of large-scale industrial production. This method increases the specific surface area of the film, making it easier to release active compounds, which is suitable for preparing films for food preservation compared to other methods.

### 4.3. Electrospinning

**Figure 3:**
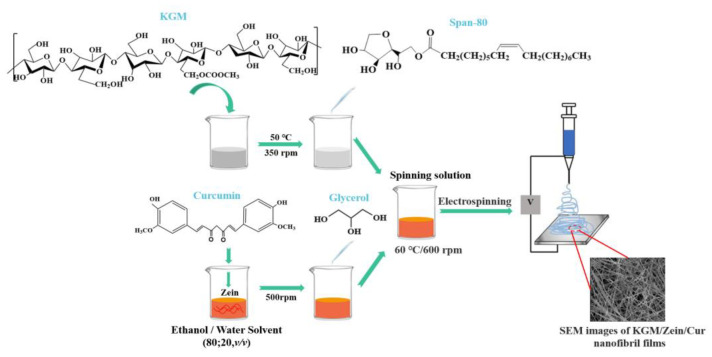
Schematic representation of preparing KGM/Zein/Cur nanofibril films via electrospinning (Adopted from Wang et al. and Li et al.) [88,90]. Nanofiber films were prepared via electrospinning of the solution loaded into a syringe covered with a 23-gauge stainless steel needle, at a controlled feed rate of 1 mL/h and an applied voltage. The nanofiber films were collected on a metal plate under conditions of 55 °C and 50% humidity.

Electrospinning involves using a strong electric field to transform droplets on the needle tip into nanoscale fiber filaments, which are then deposited on a receiver to form a nanofiber film [87]. Figure 3 presents an example preparation of an electrospinning technique [88]. This method allows for the preparation of films with nanostructures and increases the specific surface area but has disadvantages such as a low yield and strict requirements for temperature, humidity and electric field conditions. Electrospinning is primarily driven by voltage to produce KGM-based films with a nanostructure. However, this method requires raw materials with conductivity; thus, conductive spinning additives are necessary for uncharged KGM. Additionally, the yield of this method is relatively low, limiting its ability to meet demands beyond scientific research purposes [89]. 

### 4.4. Sol-Gel Conversion Compounding Method

The sol-gel conversion compounding method is commonly used to prepare biopolymer systems for coatings, especially for aquatic food preservation. This method involves converting sols into gel form and uniformly applying them onto the surface of aquatic products, similar to spraying films or coating waxes on fruits and vegetables. This not only saves space but also significantly reduces the contact between aquatic products and air, making it easier to control the quality of thin film products. To prepare biopolymer systems using this method, solutions or dispersions are first prepared, followed by casting and solvent removal [91]. For instance, Qiao et al. [92] studied the effect of κ-carrageenan addition on the properties of the agar/KGM/κ-carrageenan ternary system, particularly the microstructural changes of the resulting ternary composites at different relative humidities. The study found that the addition of a specific amount of κ-carrageenan to agar/KGM leads to the formation of ternary composite systems with enhanced properties, including an increased sol–gel transition point.

Overall, each method has its own advantages and disadvantages, and the choice of method will depend on the specific application and desired properties of the film.

## 5. Functional Properties of KGM Gel

### 5.1. Structural Stability

KGM gels possess a unique structure and texture that can be modified by adding other ingredients or changing processing conditions, making them an ideal material for loading active substances [93]. The structural stability of KGM gel depends mainly on the molecular structure and microstructure. It is necessary to characterize and demonstrate both the molecular structure (increased hydrogen bonds, formation of covalent bonds) and microstructure (observed smoother and denser structures) to prove the improved stability of the structure. Several methods, including Fourier transform infrared absorption spectrometry, X-ray diffraction, electron microscopy and atomic force microscopy, are commonly used to examine the structural stability of KGM in composite preparation [94,95].

### 5.2. Oxidation Resistance and Antibacterial Activity

Oxidation resistance and antibacterial activity are essential properties of KGM gels for food preservation. Oxidative reactions can cause irreversible damage to the flavor, appearance, texture and nutrient value of food throughout all stages of food processing and transportation [96]. Pure KGM films (i.e., KGM films without loaded active substances) typically exhibit low 1,1-diphenyl-2-picrylhydrazyl (DPPH) radical scavenging assay values due to the limited antioxidant activity of the hydroxyl groups in KGM chains. However, antioxidants such as polyphenols can enhance the scavenging of DPPH radicals in KGM films because of their antioxidant capacity and the presence of phenolic hydroxyl groups in the compounds [97]. For example, Lei et al. [98] prepared Pectin/KGM composite food packaging films loaded with tea polyphenols, which significantly increased the DPPH radical scavenging activity of the films with increasing TP content (*p* < 0.05). The control Pectin/KGM films showed a slight DPPH radical scavenging activity of 10.50%, which was attributed to the antioxidant capacity of the hydroxyl groups of pectin.

Pure KGM films lack antibacterial ability, but the addition of active substances can enhance their antibacterial properties due to several reasons. First, active substances can destroy peptidoglycan or disintegrate the outer membrane or cell wall structure of bacteria through metal ion chelation, resulting in bacterial cell death. Secondly, KGM can break existing hydrogen bonds, form new hydrogen bonds, or alter the hydrophobicity of the film, which helps balance the interactions between various compounds in the film and promote the release of active substances [99]. Thirdly, active substances can create an environment with a lower pH value that inhibits bacterial growth [100]. For instance, You et al. demonstrated that the addition of blackcurrant anthocyanin (BCA) significantly improved the antibacterial activity of pH-smart reactive fish packaging films [97]. BCA is a type of flavonoid that interacts with the lipid bilayer and functions as an antibacterial agent. None of the three types of membranes (KGM, carboxymethylcellulose and carrageenan) exhibited notable antibacterial activity in the absence of BCA. However, as the amount of BCA increased, the antimicrobial activity of the KGM/carboxymethylcellulose/BCA composite membrane gradually improved.

### 5.3. Sustained Release Capability

Achieving the sustained release of antimicrobial substances from packaging films to the interior is crucial for prolonging the shelf life of aquatic foods. Composite films made by integrating active substances into KGM gel are known to exhibit sustained release during food preservation. A good sustained release capability is typically characterized by an initial rapid release, followed by a sustained slow release and, finally, plateauing. Sustained release is more stable and slower because additional time is required to break hydrogen bonding interactions and compact network structures. For instance, Wu et al. [101] prepared KGM composite films loaded with beet anthocyanins and evaluated their sustained release capability. The burst release was attributed to the red cabbage anthocyanins (RCA) on and near the film surface, which were immobilized in the inner core of the carrageenan matrix. The main mechanism of RCA release from the films is Feiffer diffusion, and the diffusion control from the films facilitates sustained antioxidant activity, antimicrobial activity and pH-sensing. Because of the long diffusion pathway, the RCA immobilized within the carrageenan matrix required more time to release and showed a gradual increase in cumulative release. Additionally, the higher RCA content led to a higher cumulative release under the same conditions, suggesting that the release of RCA from the KGM/oxidized-chitin nanocrystals/RCA matrix was mainly driven by the diffusion force caused by the change in the incorporated RCA content.

### 5.4. Permeability

#### 5.4.1. Water Vapor Permeability (WVP)

The shelf life of aquatic foods depends on their moisture content [102]. Reducing the moisture content can decrease the moisture exchange between food and the storage environment, thereby slowing down the rate of deterioration and extending the storage life of food [103]. Thus, food preservation necessitates packaging films with low water vapor permeability (WVP).

The addition of new materials to composite membranes typically decreases their water permeability due to the enhanced hydrogen bonding between KGM and other compounds [104]. Higher compound concentrations result in the improved hydrophilic tendency of the composite films. However, when the compound concentration exceeds a threshold, the excess compound cannot be uniformly dispersed, leading to a decrease in the network density of the membranes and an increase in WVP [105]. Alternatively, the decrease in the moisture content of KGM composite films may also be attributed to the hydrogen bonding interaction between KGM and other compounds in the composite membrane matrix [106].

For instance, Sun et al. [75] demonstrated that the addition of EGCG could reduce the WVP and moisture content of a KGM/carboxymethyl chitosan bio-composite film. The reduction in WVP may be attributed to the presence of strong hydrogen bonds between KGM, KGM/carboxymethyl chitosan and EGCG in the matrix. Excess EGCG reduced WVP by cross-linking polymer molecules, promoting their reassembly and subsequently binding competitively to biopolymers’ -OH, -NH2 and -COOH bonds.

#### 5.4.2. Oxygen Permeability (OP)

Oxygen permeability (OP) refers to the ability of a film to block oxygen, which is crucial for preventing food oxidation and quality deterioration [98]. Compared to other films, pure KGM film usually exhibits lower OP, and the composite KGM film has even lower OP due to its compact structure, especially the hydrogen bond cross-linking network composed of hydrogen. For example, Liu et al. [107] investigated the effect of adding emulsions to pure KGM films with a 100% (*w*/*w*) emulsion/KGM ratio and found that the resulting emulsion films had lower OP. This is attributed to the formation of an ordered hydrogen bonding network structure in the composite films after the addition of emulsions.

### 5.5. Mechanical Properties

Good mechanical properties are essential to ensuring the effective and continuous utilization of aquatic foods packaging films. To better protect aquatic products, preservation films must possess a certain level of tensile strength to prevent mechanical damage during transportation and handling. Additionally, these films should also have a certain degree of flexibility to prevent the problem of brittle fracture, which can result in the contact of air or pollutants with the aquatic products and lead to spoilage. The mechanical properties of KGM composite films are characterized by their tensile strength and elongation at break, which reflect their fracture resistance and toughness, respectively [74]. There are two ways to enhance the tensile strength of a film: (1) by incorporating materials with a higher tensile strength or (2) by increasing the interactions between materials through non-covalent and covalent bonding [77]. Composite KGM films exhibit great tensile strength (and elongation at break) because the incorporation of the composite fills the free volume between the polymer chains, strengthening the intermolecular forces. For example, Duan et al. [108] prepared κ-carrageenan/KGM/TiO_2_ nanocomposite films and found that, compared with pure κ-carrageenan/KGM composite films, the k-carrageenan/KGM/TiO_2_ (5 wt%) nanocomposite film had better mechanical properties, with a 73.1% increase in the tensile strength value and an 11.6% decrease in the elongation-at-break value.

In addition to the above-mentioned properties, during the KGM gel preparation, other measurements should be taken to ensure the characteristics and properties of the composite film, including thickness, color, transparency, swelling degree and water solubility, moisture absorption, oxygen transmission rate, carbon dioxide transmission rate and rheological properties of the film-forming solutions [109]. 

## 6. Application of KGM Gel in Aquatic Foods

KGM gel has several applications in aquatic foods preservation. One of its main functions is as a thickening agent, which can improve the texture and mouthfeel of food products [28]. KGM gel can also be used as an active packaging material for aquatic foods. KGM-based films have been shown to be effective at inhibiting the growth of spoilage bacteria, reducing lipid oxidation and extending the shelf life of aquatic food products [110]. Additionally, KGM can be used as a carrier for bioactive compounds, such as antioxidants, in aquatic food preservation. The three-dimensional gel network structure formed by KGM gelation is an excellent carrier for small active molecules and can be used as a gel carrier for loading antioxidant molecules for seafood preservation [32]. 

Meanwhile, to address the shortcomings of food preservation films, such as weight loss during temperature changes and inadequate antibacterial performance, the blending of polymers, nanoparticles and other materials has been proven to be effective [111]. Hybrid films require sufficient compatibility between their components to facilitate inter-chain interactions that result in a uniform and dense film. Biomacromolecules, such as ethyl cellulose, whey protein isolate, chitosan and conjugated cold gels, nanomaterials, such as zinc oxide nanocrystals, chitin oxide nanocrystals and cellulose nanofibers, and natural plant extracts are commonly used blending materials [112].

Polyphenols, such as catechins, anthocyanins and tannins, are active compounds with numerous health benefits for humans. They have been shown to strengthen blood vessel walls, promote gastrointestinal digestion, lower blood lipids and increase body resistance. Moreover, polyphenols are known for their strong antioxidant and antibacterial effects [113]. The antioxidant properties of polyphenols are due to the specific hydrogen donors of the phenolic hydroxyl groups on the B and C rings, which consume free radicals of fatty acids, interrupting auto-oxidation reactions and achieving antioxidation. Furthermore, polyphenols activate antioxidant enzymes, inhibit the activity of oxidative enzymes (such as complex metal ions) and eliminate lipid radicals, hydroxyl radicals and singlet oxygen to achieve antioxidation. The antimicrobial properties of polyphenols include inhibiting the attachment of pathogenic microorganisms on food, restraining the activity of microbial-related enzymes and disrupting microbial cell structures, such as bacterial cell walls and cell membranes. Additionally, KGM composite membranes are suitable for loading polyphenols for food preservation due to the good compatibility between polyphenols and KGM [75,76]. The application of KGM gel in aquatic foods will be presented, with a particular focus on KGM-based sustained release gels, films and composite coating.

### 6.1. KGM-Based Sustained Release Gel Loaded with ECGC

Taking inspiration from sustained release drug delivery systems, a topological structure-based sustained release gel carrier has been developed for food preservation [114]. The topological structures can reduce the molecular weight of KGM by breaking down long molecular chains into shorter ones, which in turn reduces the steric hindrance of the network system and ensures the sustained release of the compounds loaded within the gel. In a study by Wang et al. [115], a KGM-EGCG microgel was created to inhibit blackening and increase the shelf life of prawns (*Litopenaeus vannamei*). The effectiveness of the KGM-EGCG topological microgel in inhibiting prawn blackening was evaluated based on parameters such as total volatile basic nitrogen content (which reflects the volatile compounds released after the decay of shrimp), pH value, sensory evaluation, K value (the ratio of the HxR+Hx amount to the ATP amount) and PPO activity (polyphenol oxidase, an enzyme involved in shrimp blackening). The results showed that the KGM microgel could effectively embed EGCG while enhancing its stability, resulting in a sustained release effect. The KGM-EGCG microgel also exhibited stronger anti-PPO activity and helped to maintain the quality of prawns while inhibiting bacterial growth.

### 6.2. KGM-Based Films Loaded with Anthocyanins

Anthocyanins, the largest group of water-soluble natural phenolic pigments, are commonly found in fruits and vegetables and have been used to develop pH-sensitive films for smart food packaging due to their sensitivity to various pH levels, antimicrobial properties and non-toxic nature [116].

**Figure 4:**
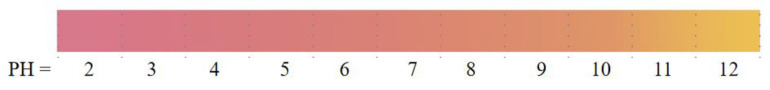
Color change of packaging film under different pH conditions [118].

Smart packaging films offer additional functionalities that can extend the shelf life of aquatic foods when compared to traditional packaging. Smart packaging is essential for dealing with external stimuli such as pH, temperature, light and biomolecules that can affect the quality and safety of food products [117]. In aquatic foods, spoilage is associated with the decomposition of proteins and the formation of alkaline substances, such as ammonia, indole, trimethylamine and histamine. This spoilage increases the pH value of aquatic foods, making pH-sensitive films an effective tool for monitoring the quality and safety of food products (Figure 4).

For example, Sun et al. [76] developed a film based on KGM loaded with chitosan, mulberry anthocyanin extract (at least two organics) and Zinc Oxide Nanoparticles, which was tested for antibacterial activity against *Escherichia coli (E. coli)* and *Staphylococcus aureus (S. aureus)*. The results indicated that the inclusion of nanomaterials increased the specific surface area and network densities of the films, thereby protecting and improving the sustained release of anthocyanins. Wu et al. [118] developed a novel intelligent film by immobilizing 1%, 3%, and 5% black rice bran anthocyanins (BACNs) into the oxidized-chitin nanocrystals/chitosan matrix. The ultraviolet-visible spectrum of BACNs solutions showed color variations from red to greyish green in a range of pH 2.0–12.0. The results showed that the developed films could be used as intelligent food packaging for monitoring animal-based protein food spoilage. Zhou et al. [78] prepared pH-sensitive food packaging films based on KGM and hydroxypropyl methyl cellulose (HPMC) incorporated with mulberry extracts (MBE). Experiments showed that the transparent films wrinkled with the spoilage of silver carp after 24 h. After 36–48 h of fish spoilage, the films of KH-MBE-5% changed slightly from gray to yellow, the films of KH-MBE-10% showed a dark brown color and the films of KH-MBE-20% showed almost no change. The results indicated that the KH-MBE-20% film had the best preservation effect due to the increased MBE concentration. For all experimental composite films, the best antioxidant and antibacterial properties were achieved when the MBE concentration was 20%, and they have the potential to be applied in the real-time monitoring of fish freshness. Cao et al. [119] developed a KGM-based film reinforced with pullulan and acai berry extract using the solvent casting method. The film exhibited pH-sensitive properties and has potential applications for fish freshness detection. You et al. [97] found that blackcurrant anthocyanins could improve the structure and properties of KGM/carboxymethyl cellulose composite films. The composite film is a biologically active smart packaging film with a pH response, antibacterial and antioxidant activities and the ability to detect fish freshness.

### 6.3. KGM-Based Composite Coating

A composite coating for frozen aquatic foods is a novel form of film that incorporates natural ingredients [120]. The film is formed by immersing the aquatic food in a pre-prepared solution with air bubbles removed, freezing the food at low temperatures and forming the packaging film. This type of film is edible and non-toxic, providing an advantage over traditional packaging methods [121]. The composite coating film adheres closely to the surface of the aquatic food, creating a tight seal around the product and packaging, thereby reducing exposure to air and inhibiting oxidation and spoilage. Additionally, the close contact between the coating film and the aquatic food’s surface increases the contact area, allowing antioxidant active molecules in the film to effectively preserve the product’s freshness. 

For example, Pang et al. [122] conducted a modified atmosphere preservation method for the large yellow croaker. They immersed cleaned fish in the composite coating solution with a mass ratio of the fish body to the composite coating solution of 1:2, soaked it for 10 min and air-coated the fish body in a 4 °C ventilated room for 30 min. The fish bodies were then vacuum-packed, and a gas mixer was used to put a proportion of gas into the bags. Finally, the micro-freezing preservation of packaged large yellow croakers was combined with the modified atmosphere packaging. The experimental results showed that the composite films effectively inhibited the growth and reproduction of microorganisms and delayed the deterioration of large yellow croakers, thus extending the shelf life and enhancing economic benefits. Xiao et al. [123] developed a composite coating using KGM as the film-forming matrix and ε-polylysine hydrochloride (ε-PL) and ferulic acid as the preservative. The composite coating successfully inhibited the formation of odor compounds such as 2-nonenone, isoamyl alcohol monomer, ammonia and trimethylamine, delaying the deterioration of fish and improving freshness.

## 7. Conclusions

KGM has gained widespread popularity in various industries, including food, medicine and aquatic food preservation. This is due to its unique properties, such as its gel-forming ability, film-forming capability and water-retaining capacity. To date, the KGM-based gel carriers have demonstrated promise in improving the preservation of aquatic food. The three-dimensional gel network structure of KGM provides an effective barrier for the compounds it carries and allows for sustained release, thereby enhancing the antioxidant and antimicrobial properties of these molecules. However, further exploration is needed in several areas: (i) understanding the mechanism of interaction between KGM and active substances in the gel network, as well as the interaction between KGM and other complex polysaccharides and proteins. By studying these interactions, more suitable biopolymer materials and antioxidant active molecules can be identified, and non-covalent and covalent bonds can be rationally combined to enhance their stability further. (ii) Finding non-toxic, biocompatible and more stable synthetic materials to compensate for the low mechanical strength of KGM in order to improve the availability of KGM-based materials. (iii) Researching new methods for preparing KGM-based films, utilizing emerging technologies such as microfluidics, electrospinning, 3D printing and others, to explore more innovative and intelligent carriers. 

## Figures and Tables

**Table 1:** The research progress of the KGM hydrogen bond network [24,25,26,27,28].

Object	Research Contents	Result	Method	Reference
KGM single chain in vacuum	The influence of the degree of polymerization and substituents on dynamic conformation	The degree of polymerization affects the chain conformation and stability	Molecular dynamics	[26]
KGM segments in solution	The influence of the hydrogen bond change in the KGM chain segment on the structure and energy	A hydrogen bond is the main factor affecting the conformation and properties of KGM molecules	Molecular dynamics	[24]
KGM	The effect of pH on the types and quantities of hydrogen bonds in KGM	The gel strength of KGM is increased under alkaline conditions	Molecular dynamics	[25]
KGM chain hydrogen network	The stability of the hydrogen network of the KGM chain	Increasing the formation of hydrogen bonds decreases the energy of the acetyl system	Quantum spin model	[27]
KGM	Application of KGM in developing plant-based fish balls	KGM promotes the formation of the hydrogen bond and ordered structure	Rheological method	[28]

**Table 3:** The research progress of the KGM topological structure [8,40,41].

Object	Research Contents	Result	Method	Reference
KGM nanogel microfibril	The topological structure of KGM nanogels and nanofibers	Electrospinning improves the intermolecular hydrogen bonding and topological entanglement of KGM molecules	FT-IR, FESEM, DSC	[40]
KGM/EGCG nanofibers	Characterizing the microstructure of the nanofibers and discussing the mechanism of formation and the protective effect of KGM/EGCG nanofibers	KGM/EGCG nanofibers exhibit greater antioxidant activity than EGCG solution	Experimental and theoretical analysis	[8]
KGM-TP	The microstructure and thermal stability of KGM-TP gel	The KGM topology chain protects TP and has a high degree of release	Direct current	[41]

FT-IR: Fourier transform infrared spectroscopy, FESEM: field emission scanning electron microscopy, DSC: differential scanning calorimetry, EGCG: epigallocatechin gallate, KGM-TP: konjac glucomannan-tea polyphenols, TP: tea polyphenols.

## Data Availability

No new data were created or analyzed in this study. Data sharing is not applicable to this article.
